# Quantification and correlation of amyloid-β plaque load, glial activation, GABAergic interneuron numbers, and cognitive decline in the young TgF344-AD rat model of Alzheimer’s disease

**DOI:** 10.3389/fnagi.2025.1542229

**Published:** 2025-02-12

**Authors:** Anett Futácsi, Kitti Rusznák, Gergely Szarka, Béla Völgyi, Ove Wiborg, Boldizsár Czéh

**Affiliations:** ^1^Szentágothai Research Centre, University of Pécs, Pécs, Hungary; ^2^Department of Laboratory Medicine, Medical School, University of Pécs, Pécs, Hungary; ^3^Imaging Core Facility, Szentágothai Research Centre, University of Pécs, Pécs, Hungary; ^4^Department of Neurobiology, Faculty of Sciences, University of Pécs, Pécs, Hungary; ^5^Department of Health Science and Technology, Aalborg University, Aalborg, Denmark

**Keywords:** astrocyte, Barnes maze, cell number, cholecystokinin, hippocampus, microglia, medial prefrontal cortex, CCK+ interneurons

## Abstract

**Background:**

Animal models of Alzheimer’s disease (AD) are essential tools for investigating disease pathophysiology and conducting preclinical drug testing. In this study, we examined neuronal and glial alterations in the hippocampus and medial prefrontal cortex (mPFC) of young TgF344-AD rats and correlated these changes with cognitive decline and amyloid-β plaque load.

**Methods:**

We compared TgF344-AD and non-transgenic littermate rats aged 7–8 months of age. We systematically quantified β-amyloid plaques, astrocytes, microglia, four different subtypes of GABAergic interneurons (calretinin-, cholecystokinin-, parvalbumin-, and somatostatin-positive neurons), and newly generated neurons in the hippocampus. Spatial learning and memory were assessed using the Barnes maze test.

**Results:**

Young TgF344-AD rats had a large number of amyloid plaques in both the hippocampus and mPFC, together with a pronounced increase in microglial cell numbers. Astrocytic activation was significant in the mPFC. Cholecystokinin-positive cell numbers were decreased in the hippocampus of transgenic rats, but calretinin-, parvalbumin-, and somatostatin-positive cell numbers were not altered. Adult neurogenesis was not affected by genotype. TgF344-AD rats had spatial learning and memory impairments, but this cognitive deficit did not correlate with amyloid plaque number or cellular changes in the brain. In the hippocampus, amyloid plaque numbers were negatively correlated with cholecystokinin-positive neuron and microglial cell numbers. In the mPFC, amyloid plaque number was negatively correlated with the number of astrocytes.

**Conclusion:**

Pronounced neuropathological changes were found in the hippocampus and mPFC of young TgF344-AD rats, including the loss of hippocampal cholecystokinin-positive interneurons. Some of these neuropathological changes were negatively correlated with amyloid-β plaque load, but not with cognitive impairment.

## 1 Introduction

Alzheimer’s disease (AD) is the most common form of dementia, affecting more than 50 million people worldwide and causing severe social and economic burden (Deuschl et al., 2020; Scheltens et al., 2021). Despite extensive research on disease pathophysiology, the exact pathogenic processes are still not fully understood. One of the most influential theories on the pathophysiology of AD is the “amyloid cascade hypothesis,” which postulates that cerebral amyloid sets neurotoxic events into motion that precipitate Alzheimer dementia (Hardy and Allsop, 1991; Selkoe and Hardy, 2016). Other complementary theories emphasize the importance of chronic neuroinflammation (Kinney et al., 2018), or the progressive loss of limbic and neocortical cholinergic innervation (Hampel et al., 2018). Imbalance of other neurotransmitter systems has also been implicated to contribute to the pathophysiology, such as glutamate (Wang and Reddy, 2017) or GABA (Carello-Collar et al., 2023; Melgosa-Ecenarro et al., 2023; Tang et al., 2023). A growing body of evidence indicates that the GABAergic system is vulnerable to AD pathology as several components of the GABAergic system are reduced in patients with AD (Reid et al., 2021). A recent meta-analysis reported that patients with AD display lower GABA levels in their brain and cerebrospinal fluid, and GAD65/67, GABA_A_ receptors, and GABA transporters were also lower in the AD brains (Carello-Collar et al., 2023). Therefore, the GABAergic system has been proposed as a potential target for developing pharmacological strategies and novel AD biomarkers (Calvo-Flores Guzmán et al., 2018). In harmony with this concept, several studies reported reduced numbers of GABAergic interneurons in the hippocampus of transgenic mouse models of AD and found profound changes in cells immunopositive for parvalbumin (PV+), somatostatin (SST+), calretinin (CR+), and neuropeptide Y (NPY+) (Ramos et al., 2006; Popović et al., 2008; Takahashi et al., 2010; Stanley et al., 2012; Albuquerque et al., 2015; Giesers and Wirths, 2020; Xu et al., 2020; Ali et al., 2023), while negative findings exist as well (e.g., Sos et al., 2020). In harmony with the neuroanatomical data, electrophysiological studies have demonstrated dysfunctional GABAergic interneurons in animal models for AD (Palop and Mucke, 2016; Schmid et al., 2016; Hijazi et al., 2023; Shu et al., 2023) and altered cortical and hippocampal oscillatory network dynamics have also been reported in the TgF344-AD transgenic rat model (Bazzigaluppi et al., 2018; Stoiljkovic et al., 2019; van den Berg et al., 2023). Finally, the clinical findings are in line with these observations, as loss of PV+ and SST+ neurons has been documented in the neocortex and hippocampus of AD patients (Brady and Mufson, 1997; Waller et al., 2020).

Numerous experimental models of AD exist, and these models are invaluable tools for gaining a better understanding of AD pathogenesis and testing novel therapeutic approaches (Drummond and Wisniewski, 2017). The most widely used animal models are transgenic mice, but transgenic rat models are also available (Götz and Ittner, 2008; Van Dam and De Deyn, 2011; Novati et al., 2022) and rat models have distinctive advantages over mice (Leon et al., 2010; Cohen et al., 2013; Do Carmo and Cuello, 2013; Agca et al., 2016; Pang et al., 2022). For example, the amyloid precursor protein (APP) gene knock-in rat model for Alzheimer’s disease exhibits pathologies and disease progression resembling more closely to the human condition compared to transgenic mice overexpressing the APP gene (Pang et al., 2022). Furthermore, rats are typically more suitable for behavioral studies, as most rodent behavioral tests have been originally developed for rats enabling a more robust assessment of behavioral phenotypes in rat models (Paul et al., 2009; Novati et al., 2022). Rats are behaviorally better characterized and display a more complex behavioral repertoire than mice and as they are terrestrial, aquatic and arboreal mammals therefore, they often perform better in maze tasks assessing spatial cognition (Do Carmo and Cuello, 2013). Furthermore, transgenic rats offer great potential to read subtle and early aspects of AD pathology (Do Carmo and Cuello, 2013). Consequently, rat models have significant advantage for *in vivo* electrophysiology, neuroimaging, epigenetic and optogenetic studies, therefore represent an important asset for research on neuropathology (Do Carmo and Cuello, 2013).

Currently, one of the best rodent models to mimic the complete spectrum of Alzheimer’s disease neuropathology without insertion of a human tau transgene is the TgF344-AD rat line (Cohen et al., 2013). The TgF344-AD rat expresses human APP with the Swedish mutation and human presenilin 1 with the ΔE9 mutation on the Fischer 344 rat background and displays all major hallmarks of AD pathology, i.e., progressive amyloid deposition, tauopathy, cognitive dysfunction, neurodegeneration, and neuroinflammation with gliosis (Cohen et al., 2013; Pentkowski et al., 2018; Fowler et al., 2022; Bac et al., 2023), and most likely, these hallmarks develop sequentially over time (Chaney et al., 2021; Fowler et al., 2022; van den Berg et al., 2023; Fang et al., 2023). Overall, this model is a very attractive tool for research on AD pathophysiology and preclinical drug testing.

In this study, we performed an extensive quantitative histopathological analysis of GABAergic neurons and glial cells in the hippocampus and medial prefrontal cortex of TgF344-AD rats. Based on unbiased stereological principles, we quantified the number of PV, CR, SST, and cholecystokinin (CCK) positive interneurons, as well as Iba-1-positive cells, most of which are microglia, and GFAP-positive astrocytes. In addition to that, we assessed the number of amyloid-β (Aβ) plaques. Furthermore, we investigated adult hippocampal neurogenesis by quantifying doublecortin (DCX) positive immature neurons in the dentate gyrus. To evaluate the spatial learning capacities of the rats, we performed behavioral assessments using the Barnes maze apparatus. Our hypothesis was to find: (1) reduced number of GABAergic interneurons; (2) an increased number of glial cells; (3) impaired adult hippocampal neurogenesis; (4) the cellular changes will correlate with the cognitive abilities of the rats and with the β-amyloid load.

## 2 Materials and methods

### 2.1 Animals

TgF344-AD rats were generated on a Fischer 344 background by co-injecting rat pronuclei with two human genes driven by the mouse prion promoter; ‘*Swedish*’ mutant human amyloid precursor protein (APP) and a deleted exon 9 mutant human presenilin (*APPsw*, P*S1ΔE9*, Cohen et al., 2013). Both constructs were previously described (Jankowsky et al., 2001). Homo-and hemizygous TgF344-AD, and WT littermate rats, were bred in the animal facility of the Faculty of Medicine, Aalborg University from hemizygous founders purchased from Rat Resource and Research Center Columbia, MO, USA. Animals were group housed under a standard 12-h light/dark cycle at 24 ± 2°C with relative humidity of 50–60%. Food and water were available *ad libitum* in the home cages. Genotyping was verified by digital PCR and both sexes were included in this study, which is a common practice in experiments with TgF344-AD rats (e.g., Cohen et al., 2013; Bazzigaluppi et al., 2018; Pentkowski et al., 2018; Morrone et al., 2020). No sex differences were observed during phenotyping, therefore behavioral data were combined for the two sexes. Homozygous transgenic (TG/TG) TgF344-AD (*n* = 6) and homozygous wild-type (WT/WT, *n* = 7) littermate rats, at an age of 7–8 month, were included in the study.

Experiments complied with the ARRIVE guidelines and were approved by the National Danish Animal Research Committee (2019-15-0201-00215), adhering to the EU Directive 2010/63/EU for animal experiments.

### 2.2 The Barnes maze

The Barnes maze was applied to address hippocampal-dependent spatial reference learning and memory. The maze (Panlab/Harvard Bioscience, Inc.) consisted of a circular platform (diameter 122 cm) with 18 holes (diameter 10 cm) in the perimeter. The platform was elevated 90 cm from the floor. A dark escape box was located beneath one of the holes while the remaining holes were blinded. The platform was brightly illuminated as an aversive stimulus. The proximal surroundings of the maze were to remain constant to provide as visual cues for the rats to navigate around the platform.

### 2.3 Assessment of spatial learning and memory

Three days before experimental initiation rats were habituated for 30 min/day to stay in an empty transit cage. At day 0 rats were adapted to the maze by being placed in a transparent cylinder in the center of the platform for 30 s. and then guided to the escape box by slowly sliding the cylinder toward the escape hole. The rat was confined to the escape box for 2 min. On consecutive acquisition trials, on days 1 and 2, rats were confined to an opaque cylinder in the center of the platform for 15 s. After removing the cylinder, rats were allowed 3 min to explore the platform to locate the escape box. Each day 4 trials with 1 h interval was executed for each rat.

Short-term and long-term spatial memory was assessed using two probe trials on days 3 and 10, respectively. The escape box was replaced by a disc similar to the blinded holes. Acquisition and probe trials were recorded and subsequently latency to enter the hole, or nose poke on the disc for the first time was scored manually.

### 2.4 Brain tissue fixation and processing for immunohistochemistry

After an overdose of sodium pentobarbital (200 mg/mL dissolved in 10% ethanol), animals were transcardially perfused with 0.9% physiological saline followed by 4% paraformaldehyde (pH = 7.4). Serial coronal sections were cut throughout the entire brain using a Vibratome (Leica VT1200S). Fifty micrometer thick sections were collected in series and stored in 0.1 M phosphate buffer (pH = 7.4) with 0.5% sodium azide at 4°C until staining. Nine different primary antibodies (Table 1) were used to identify four types of GABAergic cells, astrocytes, Iba-1-positive microglia and macrophages, immature neurons in the dentate gyrus, and to visualize amyloid plaques. Samples from the wild-type and transgenic groups were always processed in parallel to eliminate any nonspecific effect of the staining procedure.

**Table 1:** Primary and secondary antibodies used in this study.

Primary Ab	Dilution	Source	Lot. No.	Secondary Ab.
Anti-Doublecortin	1: 3000	Cell Signaling Technology, Cat #: 4606	6	Biotinilated-Anti-Mouse
Anti-Parvalbumin	1: 10000	SWANT, Cat #: 235	10–11 (F)	Biotinilated-Anti-Mouse
Anti-Calretinin	1: 5000	SWANT, Cat #: 7699/3H	18,299	Biotinilated-Anti-Rabbit
Anti-Cholecystokinin 8	1: 5000	AbCam, Cat #: ab43842	GR5127-5	Biotinilated-Anti-Rabbit
Anti-Somatostatin-14	1: 10000	BMA Biomedicals, Cat #: T-4103	A18197	Biotinilated-Anti-Rabbit
Anti-GFAP	1: 10000	Novocastra, Cat #: NCL-L-GFAP-GA5	6,084,636	Biotinilated-Anti-Mouse
				Anti-Mouse Alexa 488 (A21202)
Anti-Iba-1	1: 1000	Wako, Cat #: 019-19741	LER0547	Biotinilated-Anti-Rabbit
				Anti-Rabbit Alexa 488 (AB150073)
Anti-Iba-1	1:2000	SYSY, Cat.no.: 234308	-	Anti-Guineapig Alexa 647 (A21450)
Anti-β-Amyloid	1: 10000	Biolegend, Cat #: 803002	B286878	Biotinilated-Anti-Mouse
	1: 2000			Anti-Mouse Alexa 488 (A21202)
	1: 2000			Anti-Mouse CY3 (715165)

### 2.5 Immunohistochemistry procedures

Immunolabeling of GABAergic neurons, glial cells and immature dentate granule cells was performed as previously reported (Czéh et al., 2006, 2015, 2018; Rusznák et al., 2022). Briefly, a general immunohistochemistry protocol was performed as follows: Free-floating sections were thoroughly washed and treated with 1% H_2_O_2_ for 20 min. Nonspecific binding was prevented by incubating the sections for 1 h in 5% normal goat serum (Vector Laboratories, Burlingame, CA, USA). Subsequently, the sections were incubated overnight at 4°C with various primary antibodies. After thorough rinsing, the sections were incubated with a corresponding biotinylated secondary antibody for 2 h and labeling was visualized using an avidin-biotin-horseradish peroxidase kit (1:200; Vectastaine Elite ABC Kit, Vector Laboratories), and developed with diaminobenzidine (1:200; DAB Peroxidase Substrate Kit, Vector Laboratories).

Fluorescence labeling was performed using similar procedures except that we used fluorescent secondary antibodies (Table 1) furthermore the sections were labeled afterwards with DAPI. Fluorescent samples were scanned using a Zeiss LSM-710 confocal microscope with 20× (*Z* = 1 μm; Zeiss W Plan-Apochromat 20/1.0) and 63× objectives (*Z* = 0.5 μm; Zeiss Plan Apochromat 63/1.4) at high resolution and normalized laser intensity.

### 2.6 Cell and amyloid-β plaque quantification

Two experimenters (AF and KR), who were blinded to group identification, collected data. All cell counting was performed manually using either the Neurolucida or the StereoInvestigator reconstruction systems (Version 7, Microbrightfield, Colchester, VT, USA) attached to a Nikon Eclipse Ti-U bright field microscope, using 20× and 40× objectives. Quantitative analysis was performed based on a modified unbiased stereology protocol that has been reported to successfully quantify hippocampal neurons appearing in low densities (Czéh et al., 2015). The different subtypes of interneurons were counted in a systematic manner in a complete series of 50 μm thick sections starting at a random position along the entire septo-temporal axis of hippocampal formation (from −1.80 to −6.60 relative to Bregma, according to the atlas of Paxinos and Watson, 1998). With each primary antibody, we labeled every eight serial sections from the complete series, resulting in 10–12 hippocampal sections for each antibody. We also focused on the regional distribution of the neurons; thus, cells were counted in the three main hippocampal subareas (dentate gyrus, CA2-3, CA1) separately. To quantify the GABAergic interneurons in the hippocampus, first, the contours of the different hippocampal subareas were traced under low magnification, and then the GABAergic cells were examined and counted using an objective of 20× magnification, omitting labeled profiles in the outermost focal plane. The total number of labeled neurons in the hippocampus – including both hemispheres – was estimated by multiplying the number of cells counted in every eight sections by eight. The same procedure was used to quantify *β*-amyloid plaques and to quantify DCX+ immature granule cells in the dentate gyrus.

Glial cells were counted using the stereology method (Gundersen et al., 1988) as described in detail previously (Czéh et al., 2006). Stereology was performed using MBF StereoInvestigator software (Version 7) and a Nikon Eclipse Ti-U microscope utilizing the optical fractionator probe and systematic analysis of randomly placed counting frames (size, 75 × 50 μm) on a counting grid (size of 100 × 100 μm) and sampled (25 μm optical dissector with 3 μm upper and lower guard zones) to obtain unbiased counts of Iba-1-positive cells and astrocytes. All cell counts had a Gundersen coefficient (*m* = 1) of <0.10 to ensure accuracy and consistency (Gundersen et al., 1999).

In the mPFC, we quantified GABAergic neurons and A*β* plaques using a similar systemic quantification protocol as above, and as described in detail before (Czéh et al., 2018). Cell and plaque numbers in the mPFC are presented as densities (cell number/mm^3^).

### 2.7 Statistical analysis

Results are expressed as mean ± SEM. Data analysis was performed using GraphPad Prism, Version 7 (San Diego, California, USA). β-amyloid plaque numbers and cell quantification data were analyzed using two-way ANOVA (genotype × brain area) followed by Šídák’s multiple comparisons *post hoc* test. Behavioral data were analyzed using two-way repeated measures ANOVA (time × genotype), or unpaired Student’s t-test. Correlation analysis was performed using the Pearson’s correlation coefficient. The level of significance was set at *p* < 0.05.

## 3 Results

### 3.1 TgF344-AD rats had large number of amyloid-β plaques both in the hippocampus and medial prefrontal cortex

**Figure 1:**
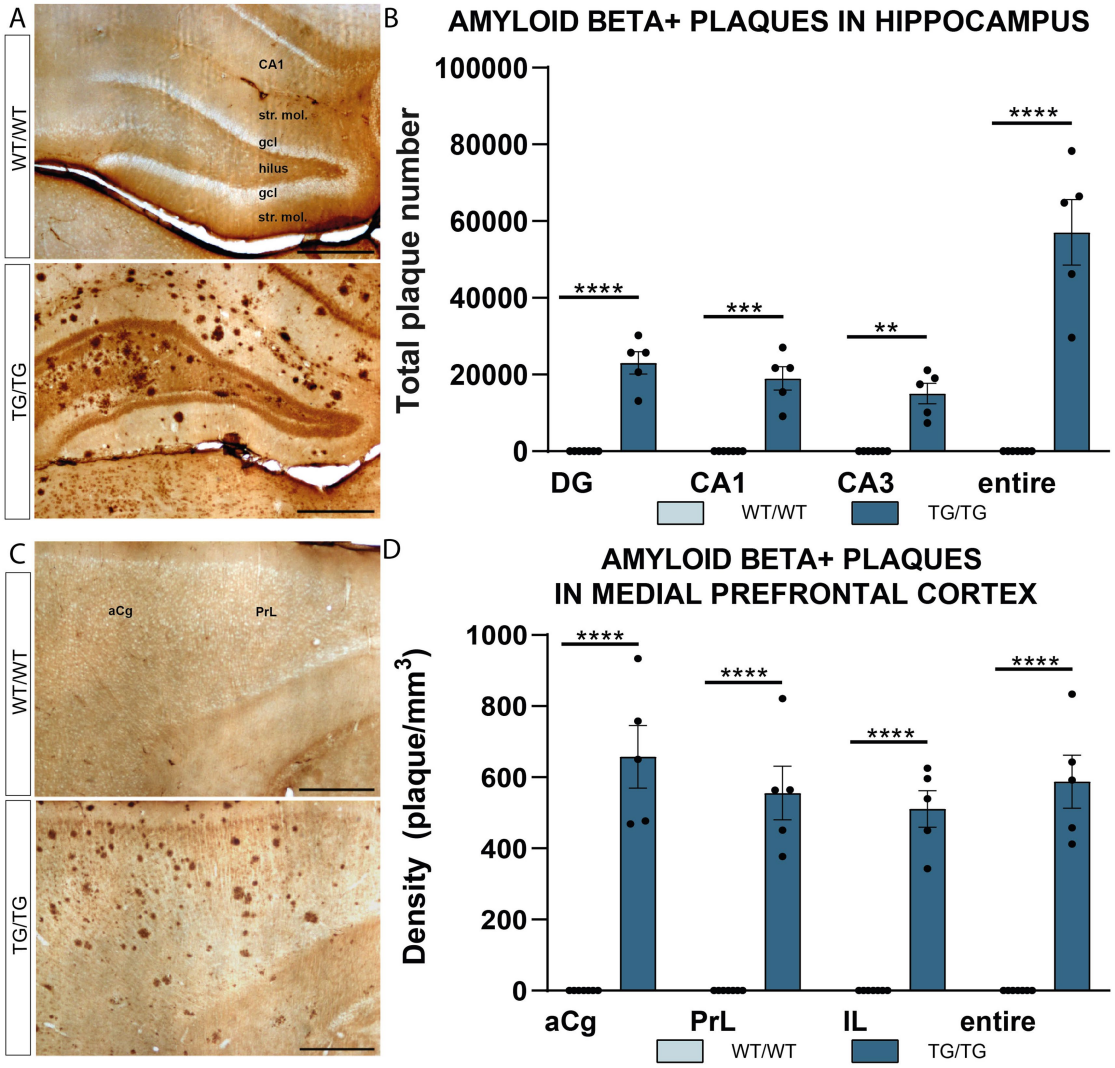
Amyloid β plaques in the hippocampus and prefrontal cortex of TgF344-AD rats. **(A)** Representative images showing immunolabeled β-amyloid plaques in the hippocampus of wild-type (WT/WT) and transgenic (TG/TG) rats. **(B)** Quantification of plaque numbers confirmed an even distribution of amyloid β plaques in all hippocampal subareas (DG, dentate gyrus; CA, Cornu Ammonis). **(C)** Aβ plaques in the medial prefrontal cortex. **(D)** Similar to the hippocampus, plaque densities showed an equal distribution in all subareas of the medial prefrontal cortex (anterior cingulate (aCg), pre-limbic (PrL), and infralimbic (IL) cortices). Statistical analysis: Two-way ANOVA (genotype × brain area) followed by Šídák’s multiple comparisons post-hoc test (****p < 0.0001). Scale bars represent 500 μm for all images.

Transgenic rats had numerous Aβ plaques in all hippocampal subareas and layers (Figures 1A,B). Similarly, in the prefrontal cortex, Aβ plaques were scattered evenly and were found in all cortical layers, all over the anterior cingulate (aCg), pre-limbic (PrL), and infralimbic (IL) cortices (Figures 1C,D). Quantification of plaque numbers revealed a significant genotype effect in both the hippocampus and neocortex. Two-way ANOVA (genotype × brain area) had significant genotype effect [*F*(1, 40) = 193.0, *p* < 0.0001] in the hippocampus (Figure 1B), as well as in the mPFC [*F*(1, 40] = 360.5, *p* < 0.0001, Figure 1D).

**Figure 2:**
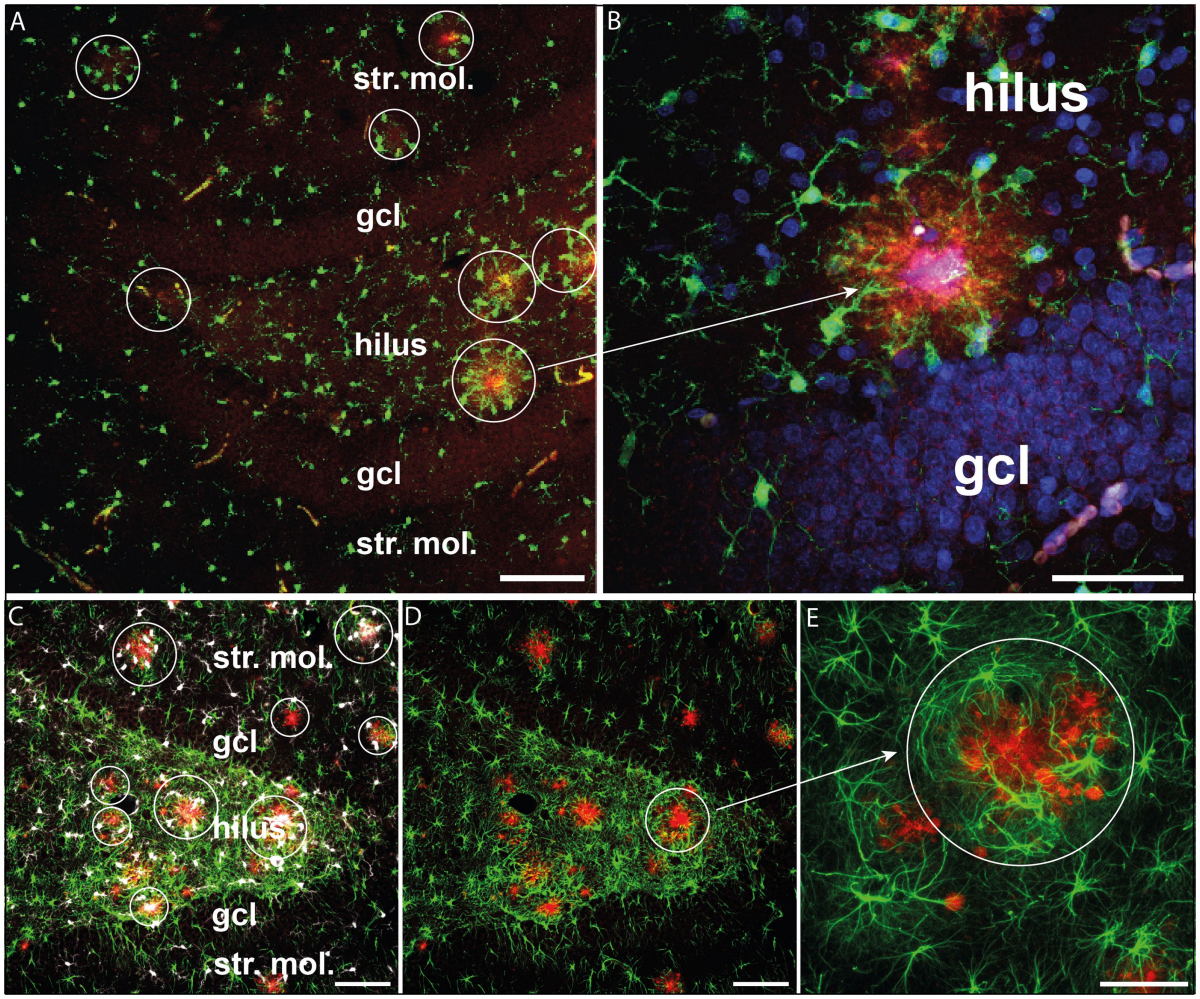
Glial activation surrounding β-amyloid plaques. **(A)** β-amyloid plaques (red) surrounded by activated Iba-1+ microglia (green) in the hippocampal dentate gyrus. **(B)** The same phenomenon is observed at a higher magnification. Iba-1+ microglial processes (green) are oriented towards β-amyloid plaques (red). Cell nuclei were labeled with DAPI (blue). **(C)** β-amyloid plaques (red) surrounded by Iba-1+ microglia (white) and GFAP+ astrocytes (green) in the hippocampal dentate gyrus. **(D)** The same hilar area where GFAP+ astrocytes surrounded the β-amyloid plaques (red). **(E)** Enlarged detail of D displaying GFAP+ astrocytes encircling the plaques (red). Scale bars: 100 μm on A, C, D, E and 50 μm on B. gcl, granule cell layer; str. mol., stratum moleculare.

We also performed fluorescence labeling and confocal microscope imaging to investigate the spatial relationship between glial cells and Aβ aggregates. As shown in Figure 2, Aβ deposits were typically surrounded both by Iba-1-positive cells and GFAP+ astrocytes. Both types of glial cells encircled the plaque deposits indicating an inflammatory response triggered by the amyloid-β plaques (Figures 2B,E).

### 3.2 TgF344-AD rats had pronounced gliosis both in the hippocampus and medial prefrontal cortex

**Figure 3:**
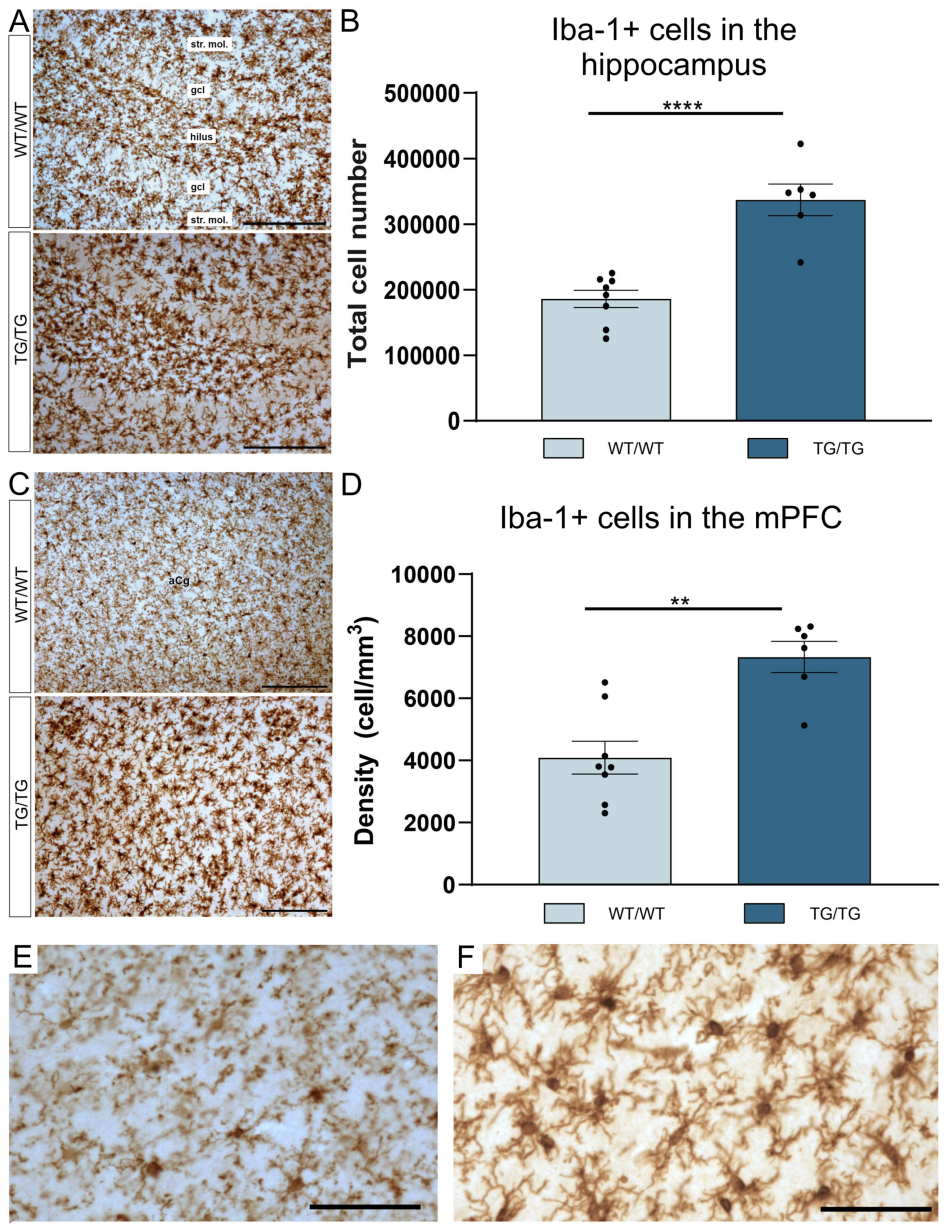
TgF344-AD rats had pronounced activation of Iba-1+ glia in both the hippocampus and mPFC. **(A)** Representative images of Iba-1+ cells in the hippocampi of wild-type and transgenic rats. **(B)** Quantitative data for Iba-1+ cells in the hippocampus. TgF344-AD rats had a significantly higher number of Iba-1+ cells in the hippocampus. Cell numbers indicate cell counts from both hemispheres. Statistical analysis: unpaired Student’s t-test; ****p < 0.0001. **(C)** Iba-1+ cells in the mPFC. **(D)** TgF344-AD rats have a significantly higher number of Iba-1+ cells in the mPFC. Statistical analysis: unpaired Student’s t-test; ** *p* = 0.001. **(E)** A high magnification image of Iba-1+ cells in the mPFC of a wild-type rat. **(F)** High magnification image of activated Iba-1+ cells in the mPFC of a TgF344-AD rat. gcl, granule cell layer; str. mol., stratum moleculare. Scale bars represent 200 μm in **(A,C)**, and 50 μm in **(E,F)**.

Iba-1+ cells in the hippocampus of wild type and transgenic rats are displayed on Figure 3A. Most of these Iba-1+ cells are microglia, although Iba-1 is also expressed by peripheral myeloid cells, such as macrophages, that may infiltrate the brain upon injury. The number of hippocampal Iba-1 cells were significantly increased [*t*(12) = 5.90, *p* < 0.0001] in the TgF344-AD rats (Figure 3B). Activated Iba-1+ cells were also seen in the mPFC of the Tg/Tg rats (Figure 3C), where these cells were present in significantly higher numbers [*t*(12) = 4.30, *p* = 0.001] compared to WT/WT littermates (Figure 3D). On the high magnification images of Iba-1+ cells (Figures 3E,F), it is clearly visible that transgenic rats had numerous activated microglia in the neocortex.

**Figure 4:**
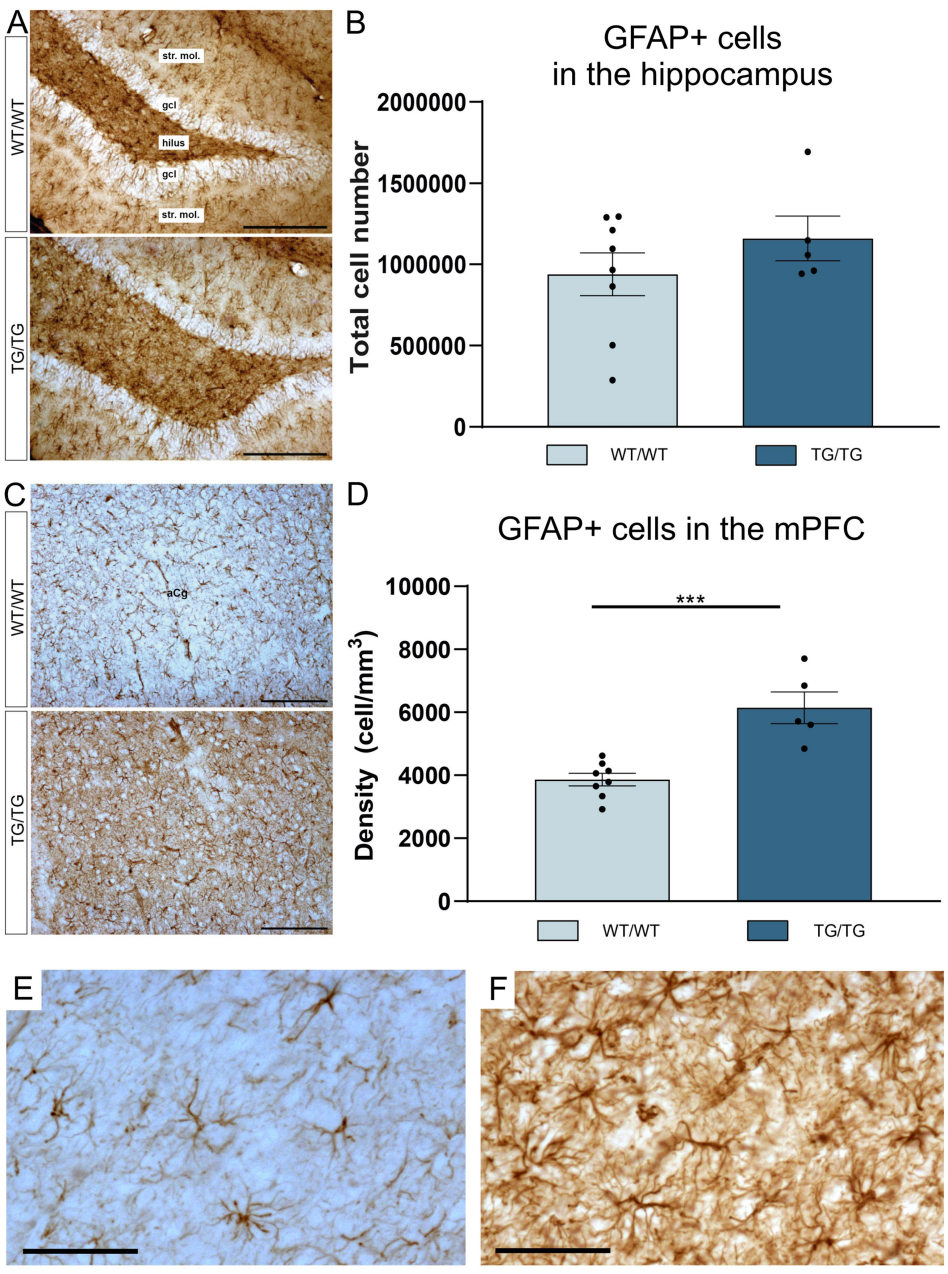
TgF344-AD rats had GFAP+ gliosis in the mPFC. **(A)** Representative images of GFAP+ astrocytes in the hippocampi of wild-type and transgenic rats. **(B)** Quantitative data for GFAP+ cells in the hippocampus. In the hippocampus, GFAP+ cell numbers were not affected by genotype. Cell numbers indicate cell counts from both hemispheres. **(C)** GFAP+ glia in the mPFC. **(D)** In the mPFC, TgF344-AD rats had significantly higher density of GFAP+ astrocytes. Statistics: unpaired Student’s t-test, *** *p* < 0.0004. **(E)** A high magnification image of GFAP+ cells in the mPFC of a wild-type rat and activated GFAP+ cells in the mPFC of a TgF344-AD rat **(F)**. gcl, granule cell layer; str. mol., stratum moleculare. Scale bars represent 200 μm in **(A,C)**, and 50 μm in **(E,F)**.

GFAP+ astrocytes are shown it the hippocampi of the WT/WT and Tg/Tg rats on Figure 4A. Results of the cell quantification indicated that hippocampal GFAP+ cell numbers were similar in both groups and were not affected by the genotype (Figure 4B). However, we found activated GFAP+ astrocytes in the mPFC of the TgF344-AD rats (Figure 4C). In the mPFC, the number of the GFAP+ cells were significantly increased [*t*(11) = 4.93, *p* = 0.0004], as it is seen on Figure 4D. High magnification images of GFAP+ astrocytes are shown in Figures 4E,F.

### 3.3 TgF344-AD rats had reduced number of CCK+ neurons in the hippocampus

**Figure 5:**
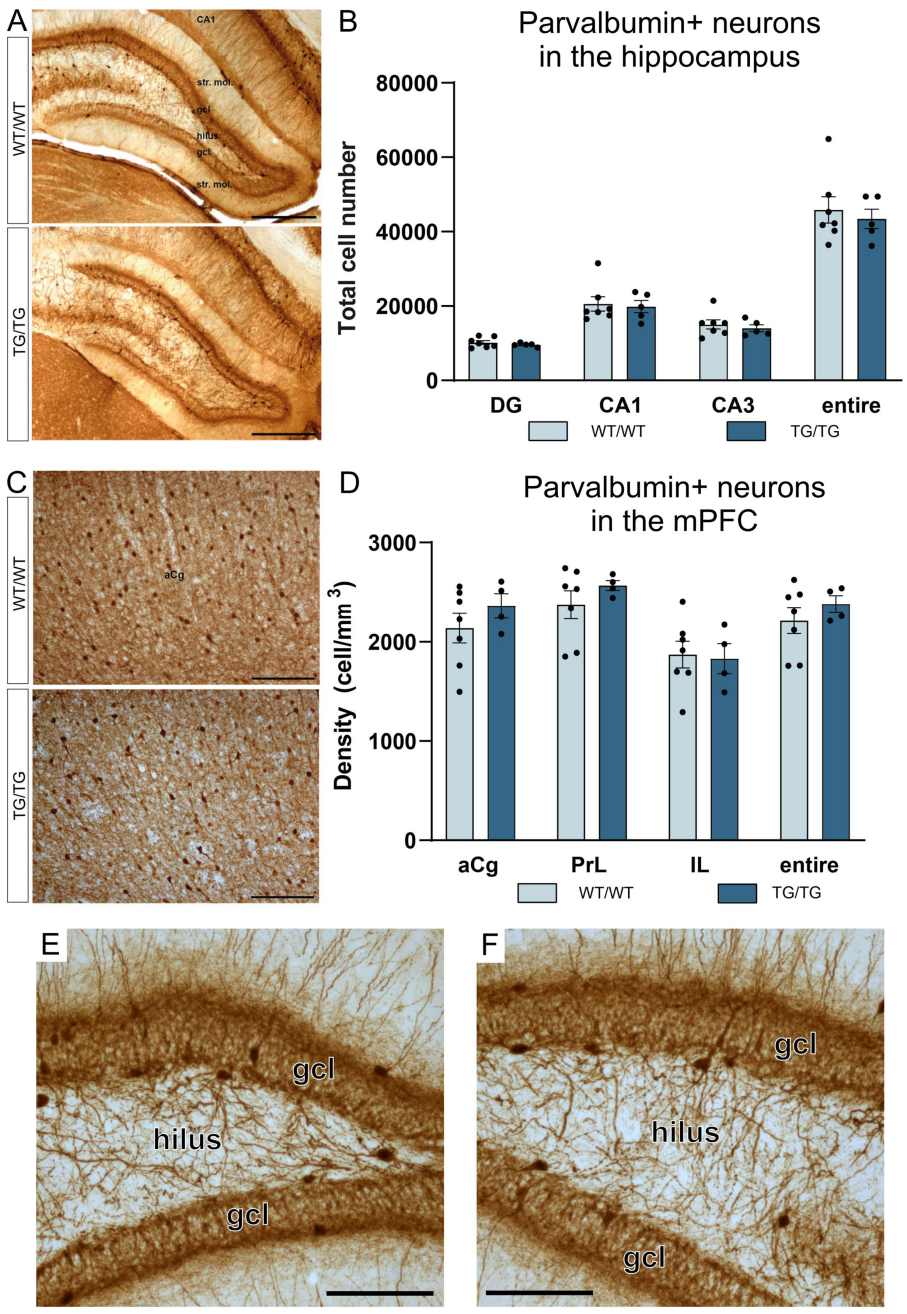
Parvalbumin-positive interneurons were not affected by the genotype. Representative images of hippocampal interneurons expressing parvalbumin **(A)**, and the corresponding cell quantification data on cell numbers **(B)**. Parvalbumin-positive neurons in the mPFC **(C)**, and PV+ cell densities in the anterior cingulate (aCg), prelimbic (PrL) and infralimbic (IL) cortices **(D)**. The number of PV+ neurons was not altered in the transgenic rats. **(E)** A high magnification image of PV+ interneurons in the dentate gyrus of a wild-type rat and PV+ cells in the dentate gyrus of a TgF344-AD rat **(F)**. DG, dentate gyrus; gcl, granule cell layer; str. mol., stratum moleculare; aCg, anterior cingulate; IL, infralimbic; mPFC, medial prefrontal cortex; PrL, prelimbic. Scale bars represent 500 μm in A, 200 μm in **(C)**, and 50 μm in **(E,F)**.

**Figure 6:**
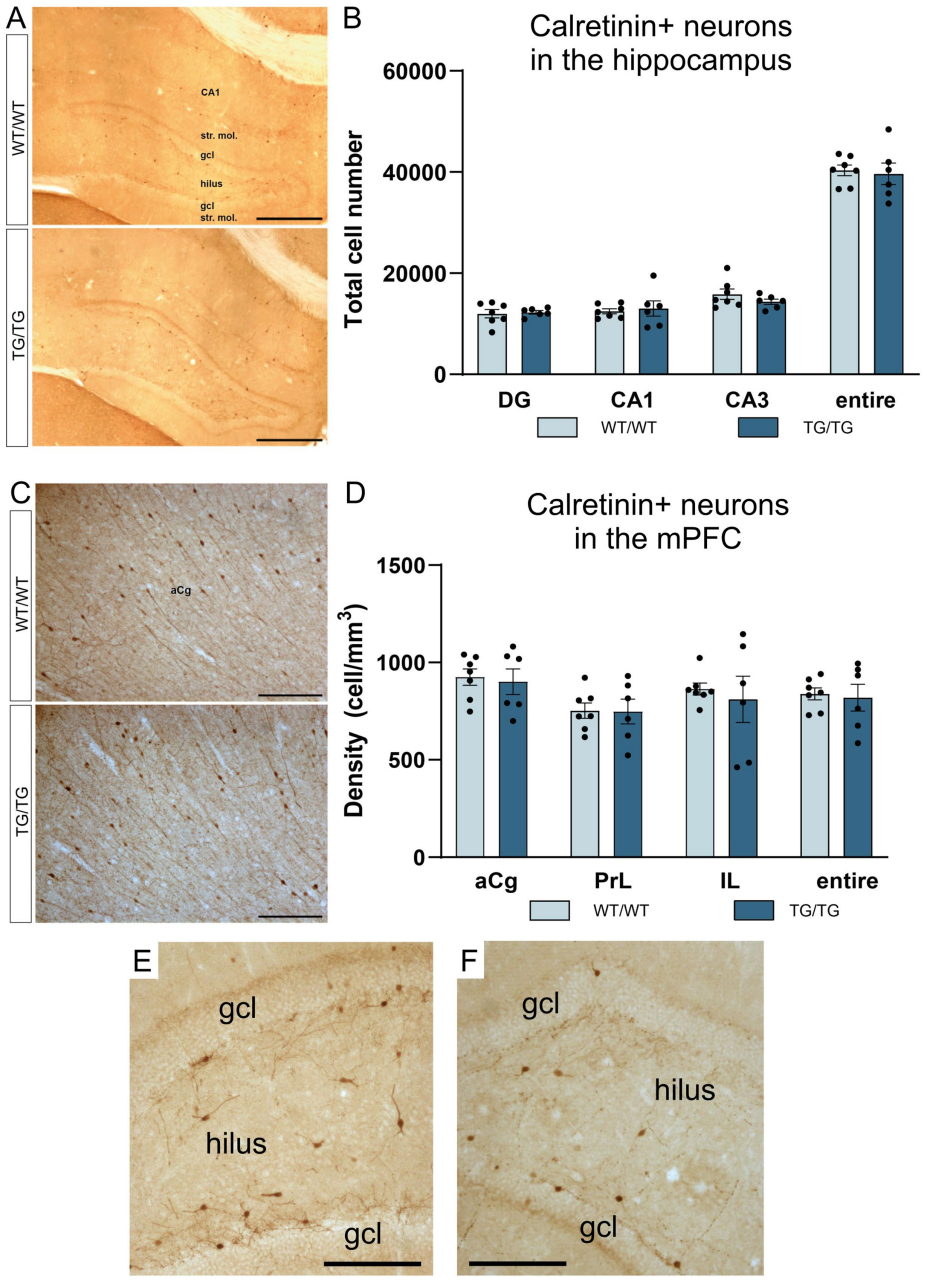
The number of calretinin-positive neurons were not affected by genotype. Calretinin immunopositive cells in the hippocampus **(A)**, and CR+ cell numbers in the hippocampal subareas **(B)**. Calretinin immunoreactive neurons in the mPFC **(C)**, and CR+ cell densities in the mPFC **(D)**. The number of CR+ neurons was not altered in the transgenic rats. **(E)** A high magnification image of CR+ interneurons in the dentate gyrus of a wild-type rat. **(F)** A high magnification image of CR+ neurons in the dentate gyrus of a TgF344-AD rat. DG, dentate gyrus; gcl, granule cell layer; str. mol., stratum moleculare; aCg, anterior cingulate; IL, infralimbic; mPFC, medial prefrontal cortex; PrL, prelimbic. Scale bars represent 500 μm in A, 200 μm in **(C)**, and 50 μm in **(E,F)**.

**Figure 7:**
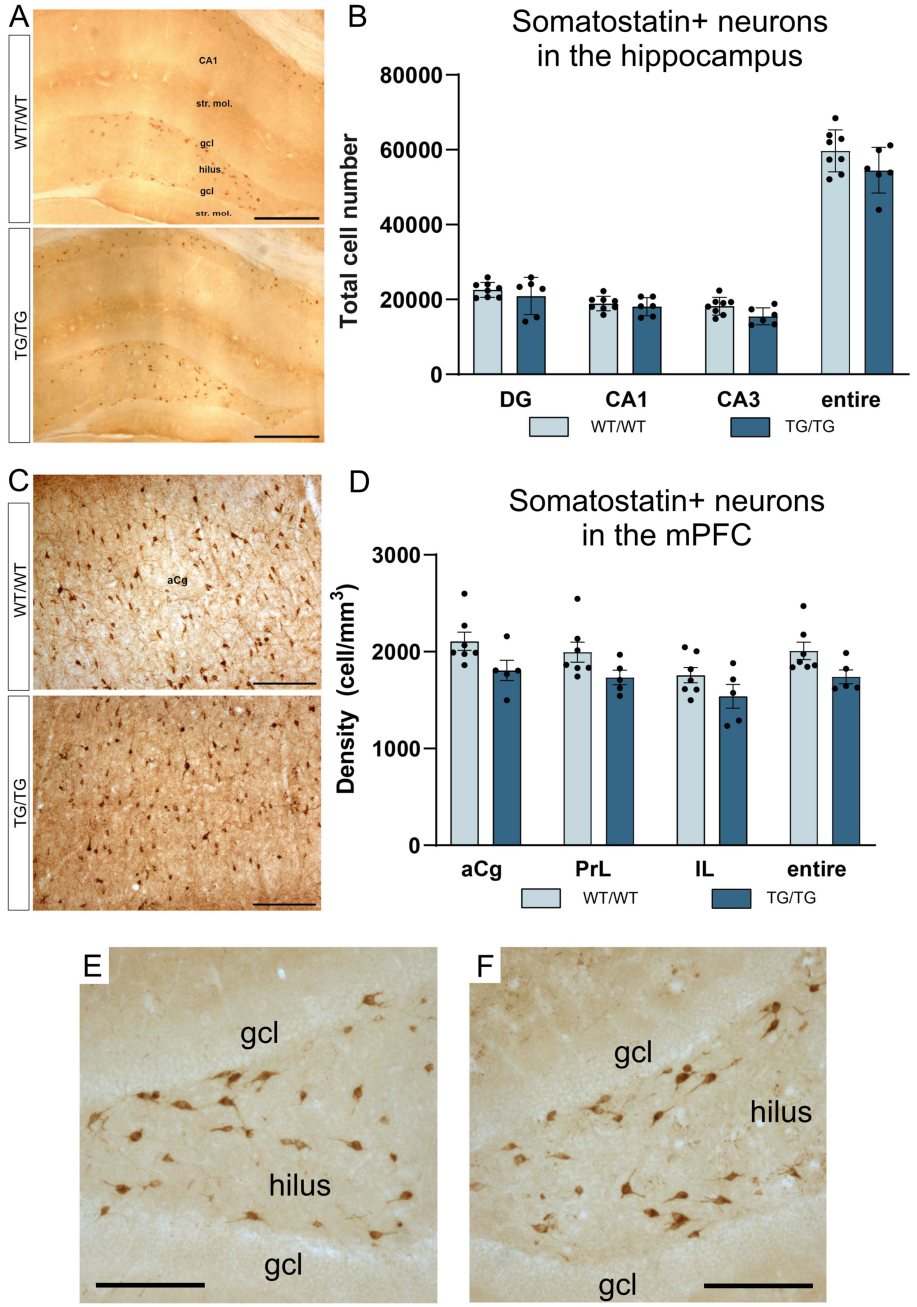
Somatostatin-positive neurons were not altered by genotype. Somatostatin-positive neurons in the hippocampus **(A)**, and the corresponding cell quantification data on hippocampal SST+ cell numbers **(B)**. Somatostatin-positive cells in the mPFC **(C)**, and a graph depicting SST+ cell densities in the anterior cingulate, prelimbic and infralimbic cortices **(D)**. The number of SST+ neurons was not altered in the transgenic rats. **(E)** A high magnification image of SST+ interneurons in the dentate gyrus of a wild-type rat. **(F)** A high magnification image of SST+ neurons in the dentate gyrus of a TgF344-AD rat. DG, dentate gyrus; gcl, granule cell layer; str. mol., stratum moleculare; aCg, anterior cingulate; IL, infralimbic; mPFC, medial prefrontal cortex; PrL, prelimbic. Scale bars represent 500 μm in **(A)**, 200 μm in **(C)**, and 50 μm in **(E,F)**.

In this study, we have quantified the number of four types of GABAergic neurons: PV+, CR+, SST+, and CCK+ interneurons because previous experiments typically report on changes affecting these cell types. We could not observe any change in cell numbers of the PV+, CR+, SST+ neurons in the young TgF344-AD rats. On Figure 5, representative images are presented for parvalbumin immunoreactive interneurons in the hippocampus and mPFC, together with the results of the corresponding cell quantification data. On Figure 6, representative images are presented for calretinin immunoreactive neurons in the hippocampus and mPFC, together with the results of the corresponding cell quantification data. On Figure 7, representative images are presented for somatostatin immunoreactive neurons in the hippocampus and mPFC, together with the results of the corresponding cell quantification data.

**Figure 8:**
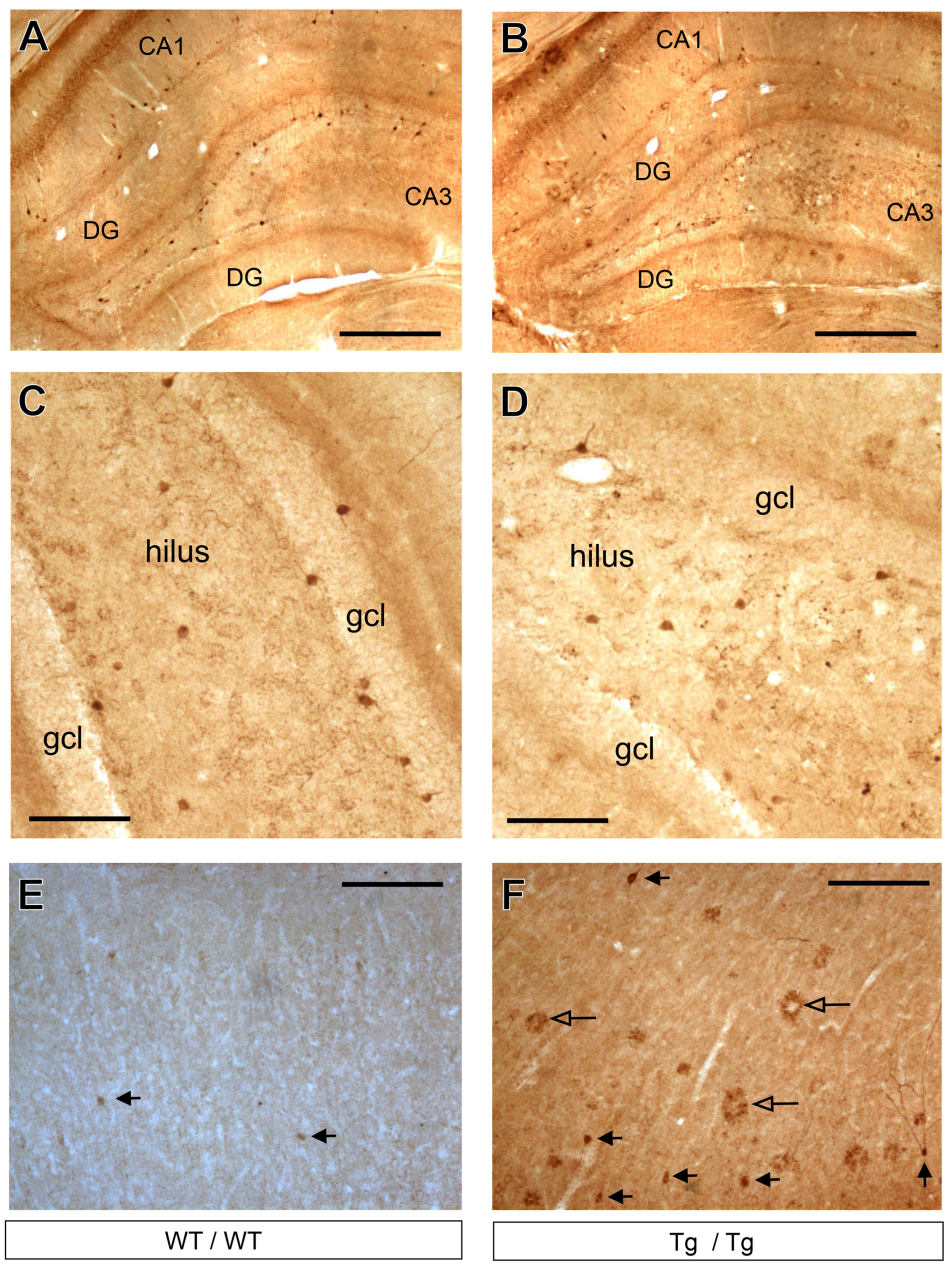
Cholecystokinin-positive neurons in the hippocampus and prefrontal cortex. **(A)** Representative images of CCK+ GABAergic neurons in the hippocampus of wild-type rats. **(B)** CCK+ interneurons in the hippocampus of transgenic rats. **(C)** Higher magnification images of CCK+ neurons in the dentate gyrus of wild-type rats. CCK + cells were clearly identifiable. **(D)** Higher magnification images of CCK+ neurons in the dentate gyrus of transgenic rats. **(E)** CCK+ cells in the frontal cortex of wild-type rats. **(F)** CCK+ neurons and CCK+ plaques in the frontal cortex of transgenic rats. Black arrowheads indicate CCK+ neurons, whereas open arrowheads point to CCK+ plaques. CCK+ plaques were present mainly in the neocortex of the transgenic rats and these objects were most likely staining artefacts. CA, Cornu Ammonis; DG, dentate gyrus; gcl, granule cell layer. Scale bars represent 500 μm in **(A,B)**, and 100 μm in **(C–F)**.

The only GABAergic cell type which was affected by the genotype was the cholecystokinin-positive interneurons. On Figure 8, we present representative images of CCK immunoreactive neurons in the hippocampus and neocortex. One should emphasize here that the visualization of the CCK immunoreaction is difficult, especially in the cortex. We have tested several primary anti-CCK antibodies before, and we decided to use the AbCam Anti-Cholecystokinin-8 primary antibody (Table 1) which gives a specific labeling and always worked reliably in our previous studies (Czéh et al., 2015, 2018; Varga et al., 2017). With this primary antibody, we could visualize CCK+ interneurons in the hippocampus of wild-type (Figure 8A) and transgenic rats (Figure 8B). This antibody typically labeled only the cell bodies and dendrites were only rarely visible, as shown in higher magnification images of Figures 8C,D. In the anterior cingulate cortex, CCK+ neurons were rather few (Figure 8E) and in the transgenic rats we could observe quite a few CCK+ plaques as well (Figure 8F). A plausible explanation for the presence of CCK+ plaques is that the CCK peptide is incorporated in the plaques, or the plaques were expressing certain epitopes which were recognized by the CCK antibody. We did not observe comparable immunoreactive plaques with any other immunostaining, only with CCK-immunohistochemistry.

**Figure 9:**
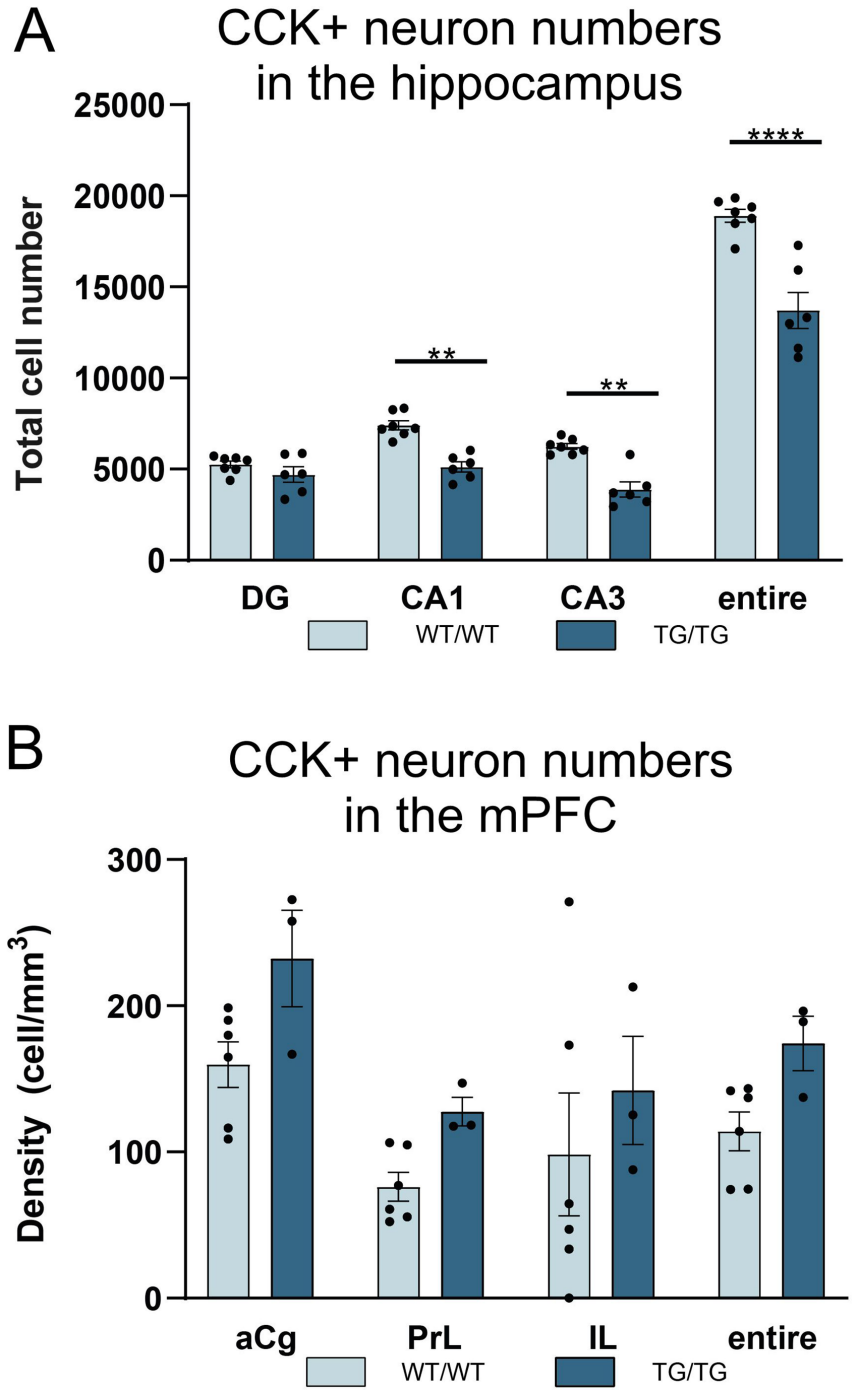
TgF344-AD rats had reduced numbers of cholecystokinin-positive (CCK+) interneurons in the hippocampus. **(A)** Quantification of CCK+ cells revealed that TgF344-AD rats had a reduced number of CCK+ cells in the CA1 and CA3 areas, as well as in the entire hippocampus. Cell numbers indicate cell counts from both hemispheres. Statistical analysis: Two-way ANOVA (genotype × brain area) followed by Šídák’s multiple comparisons post-hoc test CA1: ** *p* = 0.0014; CA3 ** *p* = 0.0020; entire hippocampus: ****p < 0.0001. **(B)** Cell quantification data revealed no genotype effect on CCK+ cell densities in the mPFC. CA, Cornu Ammonis; DG, dentate gyrus; aCg, anterior cingulate; PrL, prelimbic; IL, infralimbic.

The systemic cell quantification revealed that CCK+ neuron numbers were significantly reduced in the hippocampus (Figure 9A). Two-way ANOVA (genotype × brain area) revealed significant genotype effect [*F*(1, 44) = 72.92, *p* < 0.0001] and CCK+ cell numbers were significantly reduced both in the CA1 and CA3 subareas as well as in the entire hippocampus, but not in the dentate gyrus (Figure 9A). Pairwise comparisons using Šídák’s multiple comparisons *post hoc* test indicated that the TgF344-AD rats had significantly fewer CCK+ interneurons in the CA3 (*p* = 0.0014) and CA1 (*p* = 0.002) hippocampal subareas as well as in their entire hippocampus (*p* < 0.0001) (Figure 9A). In the mPFC, we observed a tendency for increased CCK+ cell numbers, but statistically this was not significant (Figure 9B).

### 3.4 The number of immature neurons in the dentate gyrus was not altered in the young TgF344-AD rats

**Figure 10:**
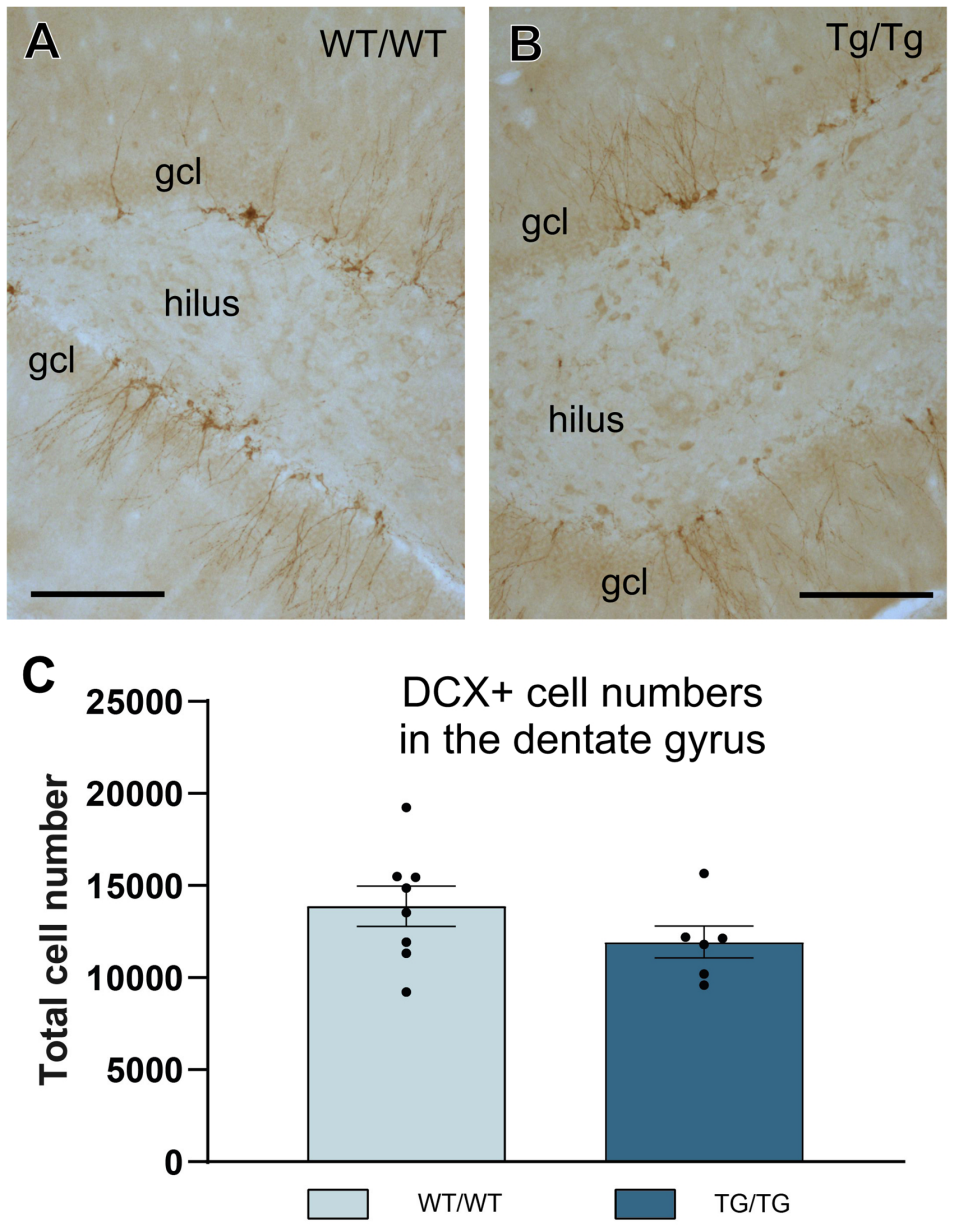
Adult hippocampal neurogenesis was not altered in the young TgF344-AD rats. **(A,B)** Representative images of doublecortin-positive (DCX+) immature neurons in the hippocampal dentate gyrus of wild-type and transgenic rats. **(C)** Systematic cell-count data. We found a small, but non-significant reduction of DCX+ cells numbers in the transgenic rats. Cell numbers indicate cell counts from both hemispheres. DG, dentate gyrus; gcl, granule cell layer. Scale bars represent 200 μm.

To quantify newly generated neurons in the adult hippocampal dentate gyrus, we used doublecortin-immunolabeling because doublecortin (DCX+) is expressed by immature neurons and therefore it is a widely used marker to assess adult neurogenesis (Couillard-Despres et al., 2005; von Bohlen Und Halbach, 2007). TgF344-AD rats had less DCX+ cells in their dentate gyrus, but this difference was statistically not significant (Figure 10).

### 3.5 TgF344-AD rats had impaired spatial learning and memory performance in the Barnes maze

**Figure 11:**
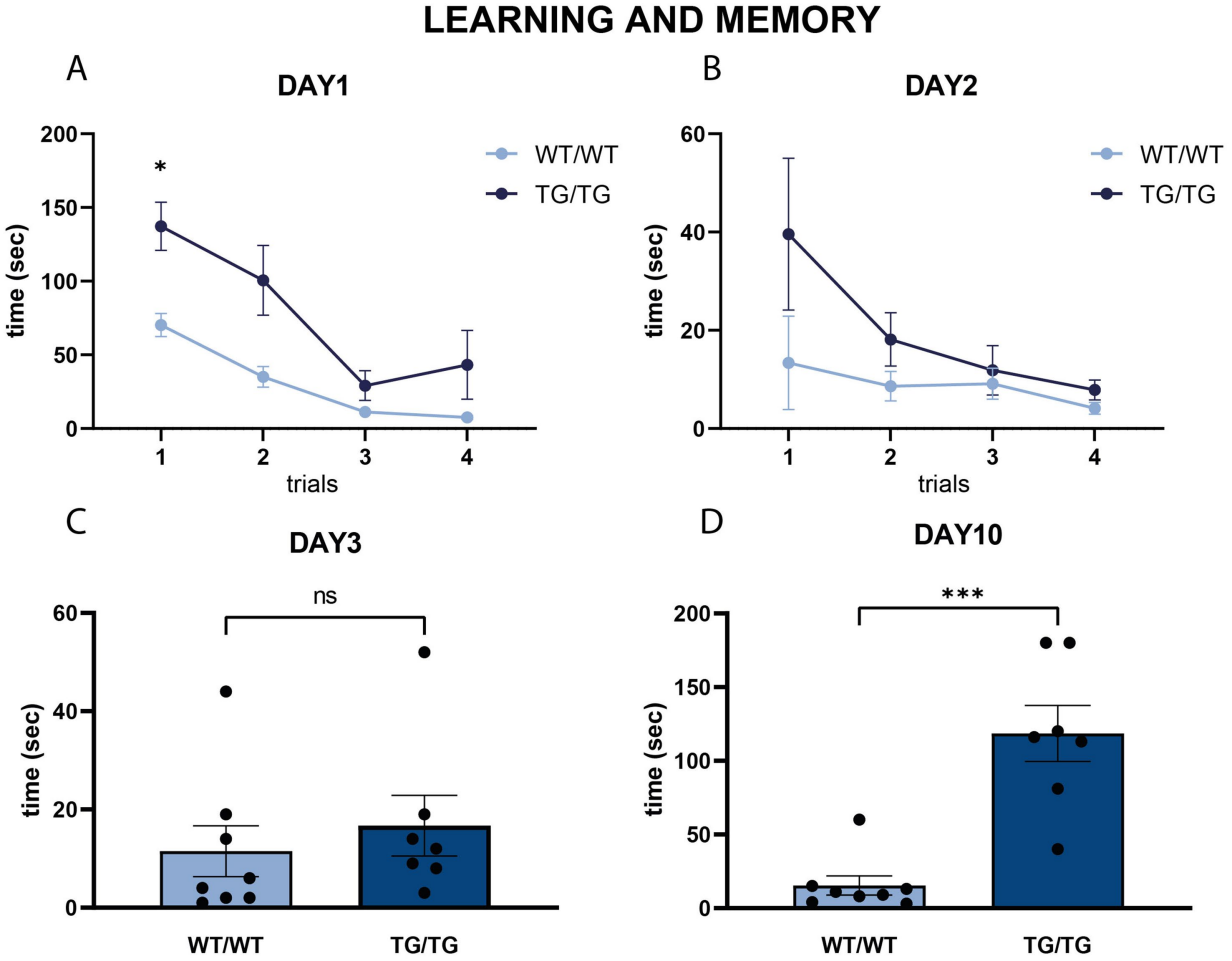
Learning and memory in the Barnes maze. **(A,B)** Line diagrams represent the learning curves in the Barnes maze task, where rats were tested in four subsequent trials on day 1 **(A)** and day 2 **(B)**. Spatial learning of the wild-type rats was significantly faster in the trials of the first day. Repeated measures two-way ANOVA (time × genotype) detected significant difference between the learning curves of the wild-type and transgenic rats on the first training day (*p* < 0.01), and Šídák’s multiple comparisons post-hoc test detected a significant difference between the first trial of day 1 (* *p* < 0.05). **(C,D)** Results of the two probe trials on Day 3 **(C)** and day 10 **(D)** indicating spatial memory competences. On Day 3, no difference was present between the WT/WT and Tg/Tg animals, but on Day 10 transgenic rats spent significantly more time finding the escape box, indicating impaired spatial memory Statistics: unpaired Student’s *t*-test, ***p < 0.0001.

On Figure 11, we present behavioral data on the performance of the rats in the Barnes maze. On the first two days, when the rats were subjected to the spatial learning trials, TgF344-AD rats were impaired finding the escape box, especially on the first day (Figures 11A,B). Repeated measures two-way ANOVA (time × genotype) revealed a significant effect of time (*F* = 20.57, *p* < 0.0001), and genotype (*F* = 16.97, *p* = 0.001). Šídák’s multiple comparisons post-hoc test detected a significant difference between the wild-type and transgenic rats in the first trial of day 1 (*p* < 0.05).

Short-term and long-term spatial memory was assessed using two probe trials of the Barnes maze on days 3 and 10, respectively. On day 3, TgF344-AD rats had similar short-term memory as the wild-type rats (Figure 11C). However, on day 10, TgF344-AD rats had a significantly impaired long-term spatial memory, as it took much longer for them to locate where the escape box was [*t*(13) = 5.42, *p* < 0.0001, Figure 11D].

### 3.6 Correlation analysis comparing behavioral performance, A*β* plaque numbers, and cellular changes in the brain

As a final step in our data analysis, we performed correlation analyses comparing the behavioral data with Aβ load, and cellular changes in the brain. Since the cognitive impairment of the transgenic rats were the most pronounced in the second probe trial of the Barnes maze, which was carried out on day-10 (Figure 11D), we took these values for the correlation analysis, but we could not detect any correlation between memory impairment and the neuropathological changes in the brain.

**Figure 12:**
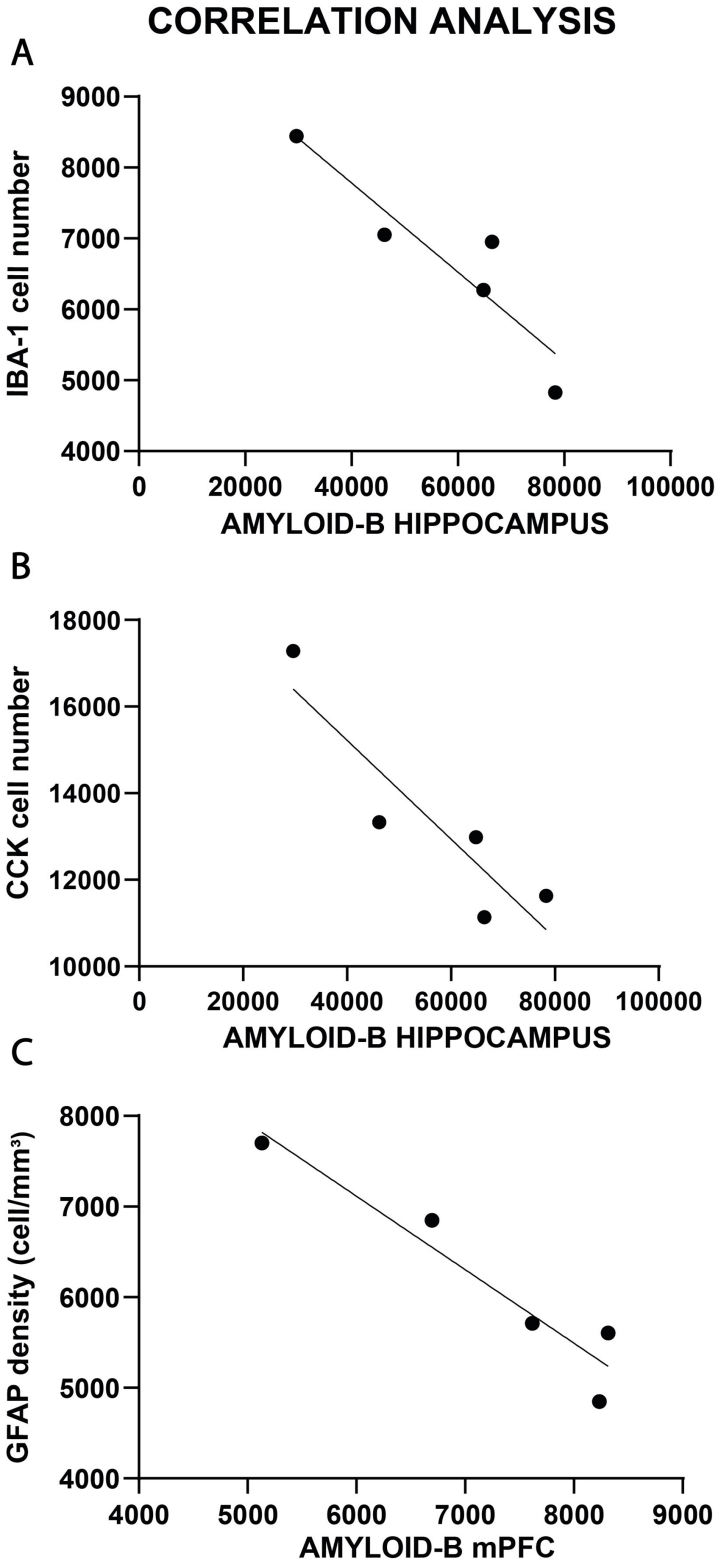
Correlations analysis between Aβ plaque numbers and cellular changes. **(A)** In the hippocampus, we found a negative correlation between Aβ plaque numbers and Iba-1+ cell numbers (*p* < 0.05). **(B)** Similarly, negative correlation was found between Aβ plaque numbers and CCK+ interneuron numbers (*p* < 0.05) of the hippocampus. **(C)** In the mPFC, there was a negative correlation between Aβ plaque density and astrocyte density (*p* < 0.05).

Correlation analysis comparing Aβ load with the cellular changes, however, yielded a few positive results which are presented on Figure 12. In the hippocampus, we found a negative correlation between Aβ plaque numbers and Iba-1+ cell numbers (Pearson *r* = −0.916, *p* < 0.05, Figure 12A), as well as a negative correlation between Aβ plaque numbers and CCK+ interneuron numbers (Pearson *r* = −0.720, *p* < 0.05, Figure 12B). In the mPFC, we found a negative correlation between Aβ plaque density and astrocyte density (Pearson *r* = −0.954, *p* < 0.05, Figure 12C).

## 4 Discussion

In this study, we report that relative to age-matched controls, young adult TgF344-AD rats have pronounced activation of glial cells in both the hippocampus and frontal cortex. We found reduced number of cholecystokinin-positive cells in the hippocampus of transgenic rats, while other types of GABAergic neurons were not affected. Young TgF344-AD rats had spatial learning and memory impairments, but this cognitive deficit did not correlate with amyloid plaque number or cellular changes in the brain. In the hippocampus, amyloid plaque numbers were negatively correlated with cholecystokinin-positive neuron, and Iba-1-positive cell numbers. In the mPFC, amyloid plaque number was negatively correlated with the number of astrocytes.

### 4.1 Glial cell activation showed negative correlation with the number of β-amyloid plaques

We observed a pronounced increase in Iba-1-positive cell numbers in both the hippocampus and the mPFC of TgF344-AD rats. This inflammatory reaction was accompanied by the activation of astrocytes, especially in the mPFC, where the number of GFAP+ cells was significantly increased. Typically, the β-amyloid plaques were surrounded by both types of glia. Several studies have documented similar pronounced gliosis in the brains of TgF344-AD rats (e.g., Cohen et al., 2013; Rorabaugh et al., 2017; Anckaerts et al., 2019; Ceyzériat et al., 2021; Bac et al., 2023; Hernandez et al., 2024). We also found a negative correlation between the number of β-amyloid plaques and Iba-1+ cell numbers in the hippocampus, as well as a negative correlation between the number of β-amyloid plaques and GFAP+ astrocyte numbers in the mPFC.

The relationship between glial activation and protein aggregation is still debated since glial cells seem to participate both in the formation and the clearing up of Aβ plaques (Nagele et al., 2004). Numerous studies performed a correlation analysis between glial cells and Aβ plaques, but the available results are ambiguous. These studies typically report that both astrocytes and microglia are spatially associated with the plaques (Serrano-Pozo et al., 2013; Dani et al., 2018), but the microglial response and the activation of astrocytes are often different and may depend on, e.g., plaque size (Serrano-Pozo et al., 2013). While some studies report on no correlation between microglia activation and Aβ load in AD patients (Arends et al., 2000; Hayes et al., 2002), a more recent *in vivo* imaging study found positive correlation between microglial activation and amyloid deposition (Dani et al., 2018). Yet another study found that brain accumulation of soluble small oligomeric species of Aβ is an early event that predates by months the classic fibrillar amyloid plaque deposition, and the amount of such oligomeric Aβ deposits showed positive correlation with GFAP-positive astrocytes in the entorhinal cortex and hippocampal CA1 (DaRocha-Souto et al., 2011).

One possible explanation for these conflicting data might be that activated microglia and astrocytes develop into heterogenous phenotypes and some may act as neuroprotective, while others neurotoxic, furthermore, their phenotypic distribution may change, based on the progression of the disease (Kwon and Koh, 2020; Gerrits et al., 2021; Ferrari-Souza et al., 2022). Regulation of Aβ levels in the brain is one of the most important functions of glial cells in AD. Currently, both microglial and astrocyte activation are regarded as a “double-edged sword,” as they actively participate in the pathogenesis of the disease, but they also play a central role as moderators of Aβ clearance and degradation (Ries and Sastre, 2016; Chun and Lee, 2018; Wu and Eisel, 2023). Importantly, both astrocytes and microglia play essential roles in phagocytosing Aβ plaques (Xin et al., 2018; Giusti et al., 2024).

Originally, we expected to find a positive correlation between Aβ load and glial activation, but to our surprise, we found the opposite. One may speculate that what we observed here was the early, acute phase of neuroinflammation, when microglial recruitment can promote Aβ clearance and hinder the pathologic progression in AD (Cai et al., 2014). Overall, our present data suggests that in the young TgF344-AD rats the presence of larger number of microglia and astrocytes was protective, as more glial cells were associated with fewer Aβ plaques, either because the glial cells were clearing them up, or because they prevented the formation of Aβ deposits. In line with this explanation, there is experimental evidence that microglia can exert neuroprotective function and clear A*β* peptides from the brain (Mandrekar et al., 2009; Fu et al., 2012; Griciuc et al., 2013). A negative correlation between microglial cells and A*β* deposits, has been shown in an experiment, where repeated injection of the macrophage colony-stimulating factor to Swedish beta-amyloid precursor protein (APP_Swe_)/PS1 transgenic mice, increased the number of microglia and decreased the number of Aβ deposits (Boissonneault et al., 2009).

### 4.2 Reduced number of cholecystokinin-positive interneurons in the hippocampus

Numerous neurotransmitter systems, including the GABAergic system, have been implicated in the pathophysiology of AD. Numerous studies have shown GABAergic neural network abnormalities in the early stages of AD, which may exist decades earlier than clinical symptoms (Palop and Mucke, 2016; Tang et al., 2023; Li et al., 2024). A recent systematic review with meta-analysis found a global reduction in various GABAergic system components in the AD brain and concluded that the GABAergic system is vulnerable to AD pathology (Carello-Collar et al., 2023). Furthermore, it has been shown that the endogenous amyloid precursor protein (APP) is highly expressed in a heterogeneous subset of GABAergic interneurons in the entire hippocampus, and during the early stages of plaque deposition, interneurons contribute to approximately 30% of the total plaque load in the hippocampus, therefore these cells are likely to have a profound contribution to AD plaque pathology (Rice et al., 2020). Based on these findings recent theories emphasize the importance of GABAergic neurons in the pathogenesis of AD and propose a concept that excitatory and inhibitory imbalance together with the structural and functional remodeling of neural networks drives the pathogenesis of Alzheimer’s disease (Bi et al., 2020; Li et al., 2024).

Many studies focusing on animal models have reported a reduced number of GABAergic interneurons in the brains of various transgenic mouse models of AD (reviewed by Xu et al., 2020). These studies typically document a pronounced reduction in the number of PV+, SST+, and CR+ neurons in the hippocampus and neocortex of transgenic mice (Ramos et al., 2006; Andrews-Zwilling et al., 2010; Baglietto-Vargas et al., 2010; Takahashi et al., 2010; Huh et al., 2016; Zallo et al., 2018; Cheng et al., 2020; Shi et al., 2020). Not only cell loss, but changes in cellular morphology have also been documented. For example, in the human amyloid precursor protein (hAPP) transgenic mouse line, a compensatory sprouting of GABAergic fibers has been found in response to the spontaneous nonconvulsive seizure activity in the hippocampus (Palop et al., 2007). In other mouse models, such as the hAPP-J20 and Mutated *Tau* VLW line, the selective loss of GABAergic septo-hippocampal axons has been observed which results in dysfunctional hippocampal network activities (Rubio et al., 2012; Soler et al., 2017). Selective degeneration of entorhinal-CA1 synapses onto parvalbumin+ neurons together with the loss of CA1 PV-positive spines have also been reported (Shu et al., 2016; Yang et al., 2018).

Human studies have also revealed changes in GABAergic cell number. In postmortem samples of AD brains, a substantial decrease in the number of PV+ interneurons has been found in all hippocampal subareas except in the CA3 subfield (Brady and Mufson, 1997). A significant loss of CR+ neurons has been reported in the dentate gyrus of AD patients (Takahashi et al., 2010). Reduced somatostatin-like immunoreactivity, potentially indicating loss of SST+ neurons, has been reported in several neocortical areas of patients with AD, including the frontal lobe (Davies et al., 1980; Candy et al., 1985; Beal et al., 1986; Mazurek and Beal, 1991).

In our present study, the transgenic rats did not have any alterations in the number of PV+, SST+, or CR+ neurons; instead, the number of CCK+ interneurons was reduced in all hippocampal areas, except the dentate gyrus. Furthermore, we found a negative correlation between the number of β-amyloid plaques and CCK+ neuron numbers, indicating that a more pronounced amyloid-β load was associated with fewer CCK+ cells.

CCK is a neuromodulator neuropeptide which facilitates hippocampal glutamate release and gates GABAergic basket cell activity, and by that modulates learning and memory. More importantly, as recent studies suggest, it seem to exert neuroprotective effects in AD (Reich and Hölscher, 2024). An early human postmortem study reported significant reduction of CCK-like immunoreactivity in several cortical areas of patients with AD, indicating that CCK+ neurons are affected by the AD process (Mazurek and Beal, 1991). Another clinical study measuring cerebrospinal fluid levels of CCK found that higher CCK was associated with a decreased likelihood of having mild cognitive impairment or AD, suggesting that CCK may serve as a biomarker of neural integrity, and cognitive performance in AD (Plagman et al., 2019). Furthermore, a growing number of evidence suggest that a CCK analog (CCK-8 L) exert neuroprotective effect in mouse models of AD. For example, in a recent study, a CCK analog effectively improved spatial learning and memory, reduced amyloid plaque load in the brain, enhanced synaptic plasticity in the hippocampus, and normalized synapse number and morphology in APP/PS1 mice (Zhang et al., 2023). In a follow-up study, the same group reported that the CCK analogue ameliorated cognitive deficits and regulated mitochondrial dynamics by activating the CCKB receptor and the AMPK/Drp1 pathway in APP/PS1 mice (Hao et al., 2024).

Animal studies have reported diminished cerebral CCK expression and the number of binding sites in the aging rat hippocampus (Harro and Oreland, 1992) and reduced hippocampal CCK mRNA levels in APP/PS1 mice, suggesting that a lack of CCK might predispose to neurodegeneration in AD (Liu et al., 2021). In a knock-in mouse model of AD (App ^NL-F/NL-F^), the CCK+ neurons showed aberrant hyperexcitability in the early stage of AD together with a gradual decline in the expression level of CCK, whereas in aged animals a significant decrease in the number of CCK+ cells was observed in the hippocampal CA1 area (Shi et al., 2020). Furthermore, this study proposed that early hyperactivity of CCK+ cells may facilitate increased Αβ cleavage and accumulation that subsequently leads to the destruction of the CCK+ cells (Shi et al., 2020). A similar cascade of cellular events may account for the decreased number of CCK+ cells found in our present study.

### 4.3 Adult hippocampal neurogenesis in AD

Neurogenesis persists in the adult mammalian hippocampus, even in humans (Boldrini et al., 2018; Tobin et al., 2019; Moreno-Jiménez et al., 2021), but it declines with advanced age (Culig et al., 2022). A growing body of evidence suggests that it is impaired in AD, and decreased neurogenesis contributes to cognitive decline in aging (Babcock et al., 2021). Reduced neurogenesis has been documented in various transgenic mouse models of AD (Babcock et al., 2021), even in young animals by the age of 1.5–3 months (e.g., Wang et al., 2004; Wen et al., 2004; Demars et al., 2010; Moon et al., 2014; Zeng et al., 2016; Scopa et al., 2020). However, other studies have reported an increased incidence of neurogenesis in AD mouse models (Babcock et al., 2021).

Human studies focusing on adult hippocampal neurogenesis in patients with AD have yielded contradictory results. The first report by Jin et al. documented increased neurogenesis in the brains of AD patients (Jin et al., 2004). More precisely, they found increased expression of immature neuronal marker proteins, such as doublecortin, polysialylated nerve cell adhesion molecule, neurogenic differentiation factor, and TUC-4, which signal the birth of new neurons (Jin et al., 2004). Another study that examined various markers expressed at different stages of neurogenesis found that markers for hippocampal stem cells decreased, while markers for cell proliferation increased, whereas markers for the differentiation/migration phase remained virtually unchanged (Perry et al., 2012). The authors concluded that neurogenic abnormalities in AD differ between various phases of neurogenesis (Perry et al., 2012). A more recent study reported marked impairment of adult neurogenesis in patients with AD (Moreno-Jiménez et al., 2019). Similarly, another study found that the number of DCX+ cells was reduced in individuals with mild cognitive impairment (Tobin et al., 2019).

Adult hippocampal neurogenesis in the TgF344-AD rat model has been investigated before. A study by Morrone et al. (2020) examined rats at 13 months of age and they found a significant reduction of adult hippocampal neurogenesis in the transgenic rats. In our case, we found a small, but statistically insignificant reduction in the number of DCX+ immature neurons in the dentate gyrus of young TgF344-AD transgenic rats. Most likely this was an early sign for an impairment which amplifies as the animals grow older.

### 4.4 TgF344-AD rats exhibited cognitive deficits during a spatial navigation task, but their behavioral performance did not correlate with the neuropathological changes

In the present study, transgenic rats at the age–7-8 months showed clear spatial learning and memory deficits in the Barnes maze. Cognitive impairment is one of the most consistent findings in TgF344-AD rats, which gradually emerge at 7–8 months and then becomes pronounced at 10–11 months of age (Berkowitz et al., 2018). This has been repeatedly demonstrated using various spatial navigation tasks, such as the Barnes maze (Cohen et al., 2013; Fowler et al., 2022), Morris water maze (Rorabaugh et al., 2017; Berkowitz et al., 2018; Bernaud et al., 2022; Kelberman et al., 2022; Bac et al., 2023), water radial-arm maze (Bernaud et al., 2022), and in an active allothetic place avoidance task (Proskauer Pena et al., 2021). Some studies have reported cognitive impairment in transgenic rats as early as four months of age (Proskauer Pena et al., 2021; Fowler et al., 2022), but negative findings on cognitive deficits are also available (for example, Pentkowski et al., 2018).

A few studies have investigated the correlation between biomarker changes in the brain and cognitive impairment of TgF344-AD rats. For example, Bac et al. analyzed proteomic differences in the dorsal hippocampal CA1 region and correlated them with learning impairments in the Morris water maze (Bac et al., 2023). They found no correlation between amyloid plaque A*β* peptide levels and learning impairment; however, soluble Aβ peptides, phosphorylated tau, and proinflammatory cytokine levels were correlated with performance in the water maze (Bac et al., 2023). Furthermore, GFAP levels, reactive astrocytes, and microglial numbers are also correlated with learning impairment (Bac et al., 2023). More recently, Hernandez et al. employed a battery of cognitive and emotional tests and correlated the performance of young adult (aged 5–7 months) TgF344-AD rats with protein markers of inflammation and AD-like pathology in the prelimbic cortex of the mPFC, basolateral amygdala, and nucleus accumbens (Hernandez et al., 2024). Transgenic rats display maladaptive decision-making, greater apathy, and impaired working memory, and numerous associations have been found between these cognitive and emotional deficits and AD-like pathology in the relevant brain structures (Hernandez et al., 2024). However, in the present study, we could not detect any correlation between impaired spatial memory in TgF344-AD rats and neuropathological changes in the hippocampus and mPFC.

### 4.5 Limitations

The present study has several limitations. We systematically quantified Aβ plaques in the hippocampus and mPFC, but this approach does not provide a precise estimate on amyloid load because the size of the plaques was rather diverse. Measuring amyloid burden at a protein level with Western blotting or ELISA and differentiating between Sarkosyl-soluble and-insoluble fractions may yield a more precise quantitative result. Another limiting factor was the relatively low number of animals involved in this study. Furthermore, tissue fixation was not sufficient in case of some rats, that explains why we had fewer number or rats in the correlation analyses compared to the rat numbers used for the behavioral experiments. Finally, we used both male and female rats in this study, since we did not detect any differences between the sexes during the behavioral, and later in the neuropathological phenotyping. This is not unusual as experiments with TgF344-AD rats often use rats with mixed sexes (e.g., Cohen et al., 2013; Bazzigaluppi et al., 2018; Pentkowski et al., 2018; Morrone et al., 2020). Even the original Cohen et al. (2013) study stated that: “We did not observe gender differences on any of the measures reported, and therefore males and females were combined for all analyses.” Since then, however, several studies reported sex differences in the behavior and neuropathology of TgF344-AD rats (e.g., Saré et al., 2020; Chaudry et al., 2022; Srivastava et al., 2023). Therefore, a study using only male or female rats would be more rigorous, and may yield different results.

### 4.6 Conclusion

We report here that in young animals, pronounced neuropathological changes are present in the hippocampus and mPFC of TgF344-AD rats, which include marked gliosis and loss of CCK+ GABAergic interneurons. These cellular changes were negatively correlated with amyloid-β plaque load, but we found no correlation between the neuropathological changes and the cognitive impairment of the animals. Dysfunctional hippocampal CCK-positive interneurons seem to contribute to AD pathophysiology and deserve further investigations.

## Data Availability

The original contributions presented in the study are included in the article/supplementary material, further inquiries can be directed to the corresponding author.
